# Chitosan/Carboxymethyl cellulose edible coating loaded with aggregation-induced emission photosensitizers: A robust yet biocompatible antimicrobial film for Litchi preservation

**DOI:** 10.1016/j.fochx.2026.104467

**Published:** 2026-09-19

**Authors:** Shuiyuan Liang, Jiahui Guo, Xiyu Jiao, Hongtao Lei, Tian Guan

**Affiliations:** Guangdong Provincial Key Laboratory of Food Quality and Safety, South China Agricultural University, No.483 Wushan Road, Tianhe District, Guangzhou 510642, China

**Keywords:** litchi preservation, Coating film, Photodynamic inactivation, Photosensitizer, Biocompatible

## Abstract

Litchi deteriorates rapidly postharvest due to high moisture, enzymatic browning, and microbes. In this work, an eco-friendly, antimicrobial aggregation-induced emission photosensitizers-loaded chitosan/carboxymethyl cellulose (AIE-CS/CMC) coating film was developed and validated to significantly delayed pericarp browning, inhibited spoilage and retained most of the nutrients, extending litchi's shelf life by approximately eight days compared with the untreated water-blank control. These effects were attributed to the robust yet biocompatible photodynamic sterilization of AIE-CS/CMC coating film, coupled with excellent gas barrier and moisture control. Importantly, this coating film killed more than 95% *Escherichia coli*, *Pseudomonas fluorescens*, *Lactobacillus acidophilus*, *Staphylococcus aureus* and *Penicillium* in suspension (10^6^ CFU/mL), with outstanding mechanical performance (6.2 MPa tensile strength and 34.3% tensile elongation), low water vapor transmission rate (0.91 × 10^−11^ g·m^−1^·s^−1^·Pa^−1^), low ultraviolet light transmission rate (46.6% at 300 nm) and high water contact angle (78.48°). This AIE-CS/CMC coating could be considered a highly efficient postharvest quality management of litchi fruits.

## Highlights

• A robust yet biocompatible antimicrobial coating film was fabricated and validated.
• The photodynamic inactivation was powered by a unique aggregation-induced emission probe.
• This coating film demonstrated excellent preservation performance in litchi fruits.

## 1 Introduction

Litchi is a tropical and subtropical fruit that has high commercial value in the international market due to its enticing bright red skin, juicy, sweet, and nutritious white transparent flesh (Chen et al., 2023). According to statistics, in China, the comprehensive loss rate during shipment and sales is usually as high as 30–50% since the peel of litchi is susceptible to enzymatic browning within 1–2 days after harvest (Yu et al., 2023). Those low-quality browned litchis will lead to unnecessary economic losses and food waste. Furthermore, the texture of litchi will be degraded by spoilage microorganisms (e.g. *Penicillium*, *Peronophythora litchi*, *Aspergillus*) (Su et al., 2023). Therefore, there is an urgent need to extend the shelf life of highly perishable litchis.

At present, the preservation treatment of litchis after harvesting includes low-temperature refrigeration (Liu et al., 2022; Wu et al., 2025), modified atmosphere packaging (Culmone et al., 2025; Passafiume et al., 2023), chemical preservatives (Shen et al., 2025; Zhang et al., 2018), plastic packaging film (Liang et al., 2024; Wang et al., 2023), etc. However, most of them are expensive and time-consuming, making it difficult to promote in the food industry. Alternatively, biomaterial-based coating film has been widely used in the storage of litchi due to advantages of low cost, simple operation, biocompatibility, and an excellent preservation effect (Jung et al., 2020; Patidar et al., 2025; Peng et al., 2025). By soaking the litchis in *Aloe vera* gel (Ali et al., 2019), chitosan (CS) (Luo et al., 2025), potato peel starch (Patidar et al., 2025) solution, or other biomaterial solutions, a thin coating film will be formed on the surface, effectively reducing water loss and controlling respiration rate to slow down litchi browning. Among them, CS is a non-toxic adulterated, antibacterial, biodegradable, and biocompatible natural polysaccharide polymer, which has been approved by the US Food and Drug Administration as a Generally Recognized as Safe material (Sharma et al., 2020). At the same time, CS has excellent film-forming properties and is commonly used in agricultural product preservation and food packaging to maintain the quality of harvested fruits and vegetables (Wei et al., 2023). However, the mechanical properties, waterproof performance, breathability and antibacterial ability of pure CS coating film are poor and insufficient, which cannot fully meet the needs of food preservation (Hassan et al., 2018). Thus, it is encouraging to integrate with other functional biomaterials or antibacterial agent to improve the film performance and maximize its antibacterial ability.

Antibacterial agents are usually incorporated in packaging films to enhance the antibacterial ability, such as antibiotics (Wang et al., 2022), essential oils (Wu et al., 2023), silver nanoparticles (Yang et al., 2023), etc. However, most antibiotics can only kill bacteria. Aloughtgur the fungus can be inhibited by essential oils or silver nanoparticles, it is reported that the original flavor of foods would be influenced by the distinctive flavorful essential oils (Lianhua and Xiangshu, 2023; Wang et al., 2021). What's more, those silver nanoparticle-based coating films may cause food safety issues (Gencag et al., 2025; Singh et al., 2026; Zhang et al., 2021). To our delight, aggregation-induced emission (AIE) materials-based photodynamic inactivation sheds bright lights on it due to highly efficient, broad-spectrum, flavorless and safe control of microorganisms (Luo et al., 2001). For example, Wang et al. reported an AIE-containing hydrogel film for the preservation of strawberries (Wang et al., 2025). Yang et al. fabricated an agar-based photodynamic composite film powered by AIE, which demonstrated acceptable performance in pork preservation (Yang et al., 2024). The rationale behind lies in the robust sterilization that induced by cytotoxic yet green reactive oxygen species (ROS) from photosensitizers when exposed to visible light (Xiao et al., 2022). More importantly, the ROS amont of AIE can be enhanced by rational structural designs, such as extending π conjugation and enhancing electron donor-acceptance (D-A) effects (Hsiao et al., 2025).

In this work, a unique AIE molecule, named TPAS-NO was designed and synthesized with extended π conjugation and strong D-A effect. Further, this highly photosensitive yet biocompatible probe was doped into chitosan/carboxymethyl cellulose (CS/CMC) mixture and formed CS/CMC/TPAS-NO coating films (Fig. 1), leading to outstanding mechanical properties, lower water vapor transmission rate as well as ultraviolet (UV) light transmission rate, and higher water contact angle compared to the pure CS coating film. Importantly, we validated that this CS/CMC/TPAS-NO coating film exhibited excellent bactericidal performance on *Escherichia coli, Pseudomonas fluorescens, Lactobacillus acidophilus, Staphylococcus aureus, and Penicillium*, which is crucial in food preserving. As what we expected, the litchis coated with CS/CMC/TPAS-NO demonstrated delayed pericarp browning, reduced water loss, diminished relative oxidase activity and high levels of soluble sugar and total anthocyanin content after eight days.

**Fig. 1:**
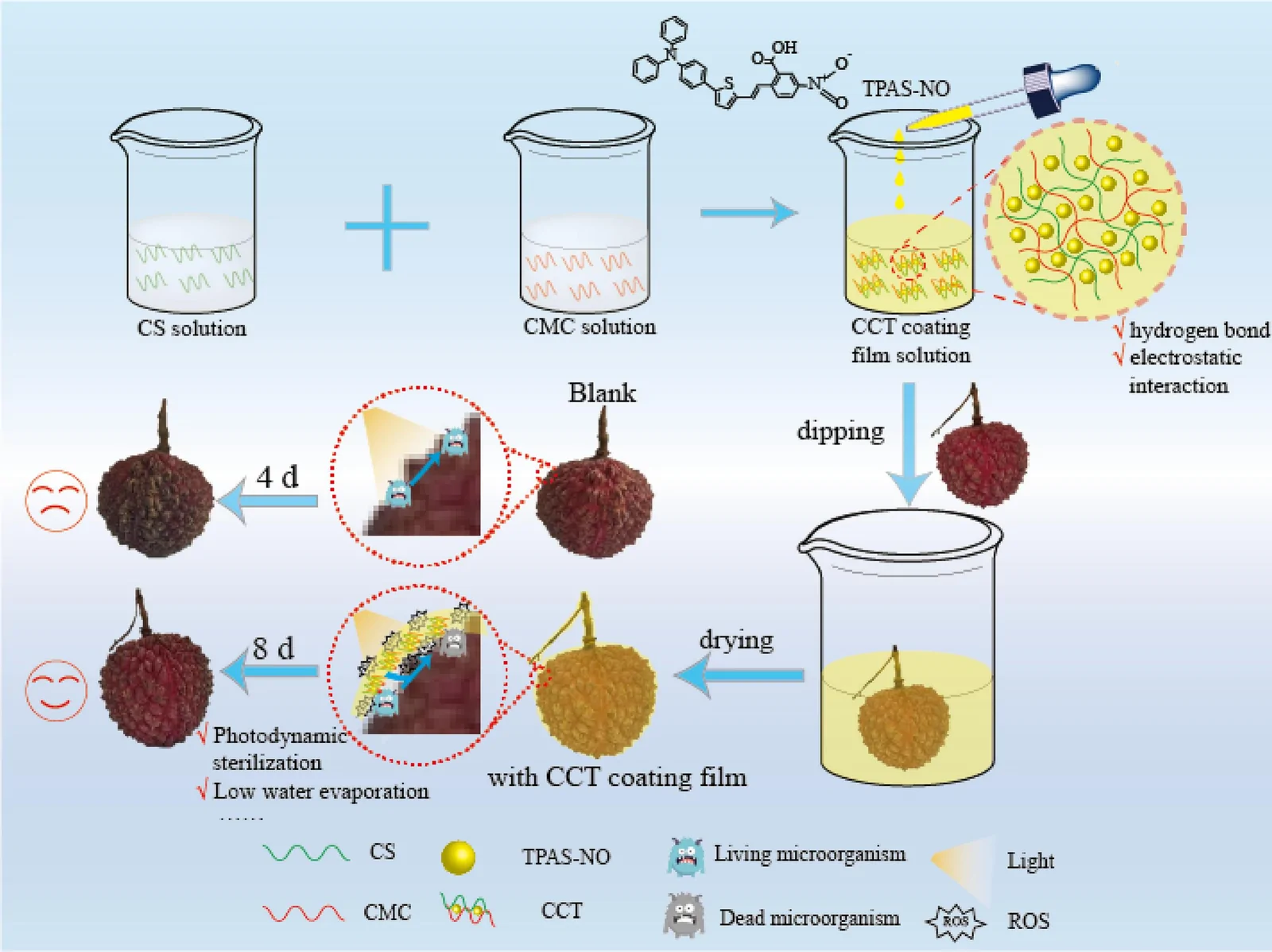
Schematic illustration of this aggregation-induced emission photosensitizers-loaded chitosan/carboxymethyl cellulose coating film for litchi preservation.

## 2 Experimental section

All the materials and instrument used in this work were listed in **Supplementary Material**.

### 2.1 Synthesis of TPAS-NO

The synthesis route of TPAS-NO was displayed in Fig. 2A, where (4-(diphenylamino) phenyl) boronic acid was utilized as a fundamental skeleton and linked with 5-bromothiophene-2-aldehyde through Suzuki-Miyaura cross-coupling reaction to get the 5-(4-(diphenylamino)phenyl)thiophene-2-carbaldehyde (2–1) product. Then, the 2–1 product was further reacted with 2-methyl-5-nitrobenzoate by Knoevenagel condensation to get the 2-(2-(5-(4-(diphenylamino)phenyl)thiophen-2-yl)vinyl)-5-nitrobenzoic acid (2–2) namely TPAS-NO. Specific procedures can be found in **Supplementary Material**.

**Fig. 2:**
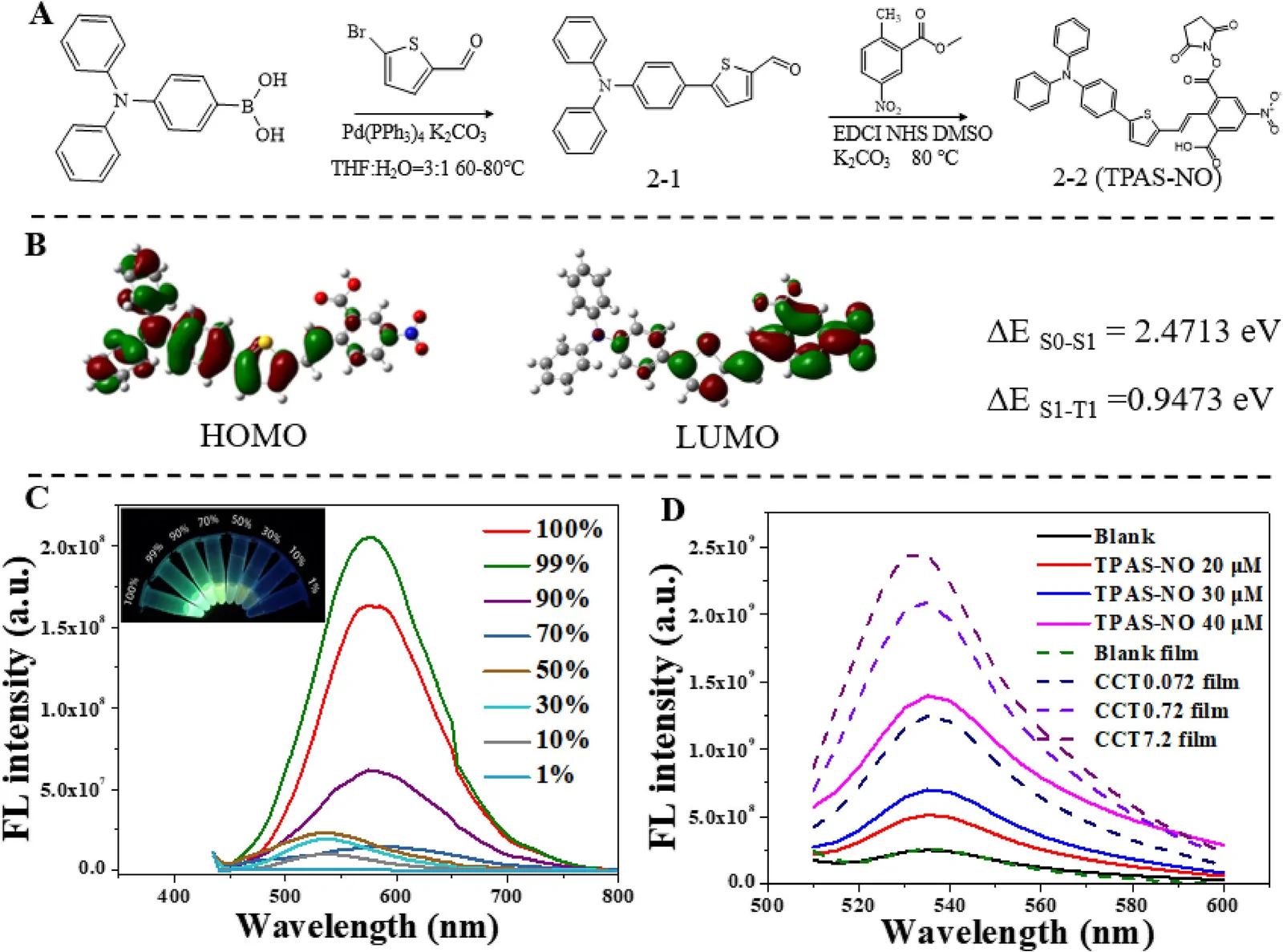
Synthesis and characterization of TPAS-NO, including the (A) synthetic route, (B) HOMO and LUMO energy gaps, (C) emission spectra of TPAS-NO in DMSO/water mixed solution system with different DMSO ratio, (D) ROS production of TPAS-NO with different concentrations in solution and coating film.

### 2.2 Optical characteristic of TPAS-NO

The fluorescence emission spectra of TPAS-NO solution (10 μM, dissolved in DMSO) at different excitation of 370, 375, 380, 385, 390, 395, 400, 405, 410, 415, and 420 nm were measured. Also, the AIE characteristic of TPAS-NO (10 μM) was explored in DMSO solutions with different water ratios (DMSO/Water, *v*/*v*, 1, 10, 30, 50, 70, 90, 99 and 100%). Their fluorescence emission spectra were collected by a UV spectrophotometer (SpectraMax i3x) under 380 nm excitation.

### 2.3 ROS production capacity of TPAS-NO

2 ‘, 7’ - Dichlorodihydrofluorescein diacetate (DCFH-DA) was used as an indicator to evaluate the ROS producing performance of TPAS-NO-D in solution and coating film (Ma et al., 2022). Detailed procedures can be found in the **Supplementary Material**.

### 2.4 Safety evaluation of TPAS-NO

To systematically evaluate the safety of CS/CMC/TPAS-NO coating film, in vitro cytotoxicity and in vivo cytotoxicity of TPAS-NO were accessed according to the responses of mouse red blood cell hemolysis, Hela cells, RAW 264.7 cells and mice. Blank group, control group (gavage with physiological saline) and TPAS-NO-treated group (gavage with TPAS-NO, 50 μM) were set, each group had four mice. The specific methodology was listed in **Supplementary Material**.

### 2.5 Preparation of coating film

First, 1 g CS (deacetylation degree: >95%, viscosity: 100–200 cps) was added to 100 mL of 1% glacial acetic acid solution and stirred at room temperature for 6 h to obtain 1% CS solution. Then, 0.5 g CMC was added to 100 mL of deionized water and stirred at room temperature for 6 h to obtain 0.5% CMC solution. Afterwards, 0.2 g glycerol was dissolved in a mixture of 1% CS solution (50 μL) and 0.5% CMC solution (50 μL), and further stirred at 50 °C for 4 h to obtain CS/CMC solution. Next, different concentrations of TPAS-NO (0.072, 0.72, 7.2, 72 μM) were added to the as-prepared CS/CMC solution and stirred for 1 h to obtain CS/CMC/TPAS-NO solution. Subsequently, 15 mL CS/CMC/TPAS-NO solution was poured into a ulture dish (diameter of 90 mm) and dried in an oven (75 °C) for 4 h. Finally, the obtained CS/CMC/TPAS-NO coating films were peeled off and kept at 25 °C (50% relative humidity) until used.

### 2.6 SEM characterization of coating film

The surface and cross-sectional morphologies of the coating films were examined using a field-emission scanning electron microscope (FE-SEM, ZEISS-GeminiSEM 360 Germany) at an accelerating voltage of 5.0 kV. For cross-sectional observation, film specimens were first cut into small rectangular pieces (approximately 5 mm × 10 mm) using a clean razor blade. To obtain a pristine fracture surface without mechanical deformation, the pieces were immersed in liquid nitrogen for at least 5 min and then immediately fractured by bending with two pairs of pre-cooled tweezers. Next, the fractured specimens were mounted vertically onto aluminum stubs using double-sided carbon conductive tape, with the cross-section facing upward. Prior to imaging, the samples were sputter-coated with a thin layer of gold (approximately 10 nm thickness) under an argon atmosphere to enhance electrical conductivity and avoid charging effects. Finally, the cross-sections were observed at a working distance of 8–10 mm using the secondary electron detector. The same preparation protocol was applied to all film samples to ensure consistency, and at least three different positions on each sample were imaged to verify the representative morphology.

### 2.7 Antibacterial performance of this CS/CMC/TPAS-NO coating film

The CS/CMC/TPAS-NO coating film was placed in 12-well plates and then 20 μL spore suspension of *Lactobacillus acidophilus, Pseudomonas fluorescens, Staphylococcus aureus, Escherichia coli* solution (10^6^ CFU/mL), and *Penicillium* were dropped onto the coating film, respectively. After irradiating with white light for 30 min, the CS/CMC/TPAS-NO coating film was transfered to a centrifuge tube containing 1 mL PBS (fungal spores were mixed with PBS containing 0.05% Tween-80) and stirred for 2 min. Next, 100 μL of these bacterial suspension at different dilution ratios was cultivated on the *Lactobacillus delbrueckii* medium (MRS), lysogenic broth medium (LB), and brain heart extract agar medium (BHI), LB medium and potato glucose agar (PDA) medium, respectively.

### 2.8 Application in litchi preservation

To investigate the preservation effect of our prepared CS/CMC/TPAS-NO coating films, fresh litchi fruits purchased from supermarket in the South China Agriculture University were employed as actual samples. In specific, these litchi samples were randomly separated into four groups, and soaked in deioned water (blank group), CS, CS/CMC (named CC group) or CS/CMC/TPAS-NO coating films with 7.2 μM TPAS-NO (named CCT7.2 group) for 2 min, respectively. Then, these treated litchi samples were dried at room temperature for 2 min and further irradiatedby white light for 20 min. Finally, these litchi samples were kept in a dark place at 20 °C. During this period, indicators including appearance changes, browning index, water loss rate, total anthocyanin content (TAC), soluble sugar content, activity of polyphenol oxidase (PPO) and peroxidase (POD) of litchi fruits were evaluated every 2 days. Each indicator was measured in triplicate. Detailed processing steps were listed in **Supplementary Material**.

## 3 Result and discussion

### 3.1 Rationale of this CS/CMC/TPAS-NO coating film in litchi preservation

Fig. 1 illustrates the preparation and application of our functional CS/CMC/TPAS-NO composite coating. The composite was formulated into a coating solution, which was then applied to litchi fruits by immersion for 2 min. Through hydrogen bonding and electrostatic interactions, a uniform thin film was formed on the fruit surface, imparting desirable mechanical strength, antibacterial properties, low moisture content, and a low water vapor transmission rate (WVTR). Subsequently, the coated fruits were illuminated for 20 min and stored at 20 °C for 8 days. Compared with the untreated water-blank control, the coated litchis showed significantly delayed pericarp browning, reduced water loss, suppressed oxidase activity, and preserved soluble sugar and anthocyanin levels, collectively extending the shelf life by approximately eight days.

### 3.2 Design and structural characterization of TPAS-NO

As shown in Fig. 2A, TPAS-NO was designed according the principles of extending the π conjugation and D-A effect, consisting of a triphenylamine moiety, a thiophene moiety and carbon‑carbon double bond and a p-nitrobenzoic acid moiety. The triphenylamine moiety serves as an electron donor and played a role in avoiding π-π stacking of TPAS-NO during aggregation. The thiophene moiety also serves as an electron donor to further enhance the overall electron supply capability, while also acts as a π bridge to provide a bridge for electron exchange between the donor and acceptor. Carbon‑carbon double bonds, like thiophene, serves as a π bridge to facilitate electron exchange between donor and acceptor. The p-nitrobenzoic acid acts as an electron acceptor to receive electrons provided by the electron donor (Kang et al., 2019). As shown in Fig. 2B, the calculation result of time-dependent density functional theory (TDDFT) was the same as what we expected, the highest occupied molecular orbital (HOMO) and lowest unoccupied molecular orbital (LUMO) has an obvious separation, leading to a low energy gap difference between singlet and triplet states (ΔE_S1-T1_ = 0.9473 eV).

Chemical structures of product 2–1 and TPAS-NO were characterized by nuclear magnetic resonance (NMR) spectroscopy. According to the ^1^H NMR spectra and carbon spectra of product 2–1 and TPAS-NO (**Fig. S1–2**), corresponding structural information can be found (detailed analysis were listed in **Supplementary Material**), suggesting the successful synthesis of TPAS-NO.

### 3.3 Physichemical properties of TPAS-NO

As shown in **Fig. S3**, the TPAS-NO solution exhibited the strongest fluorescence emission intensity at 530 nm under excitation at 380 nm. The AIE characteristic of TPAS-NO was investigated in a mixed solution of DMSO/water (*v*/*v*). As the proportion of DMSO increased, the fluorescence gradually enhanced (Fig. 2C) from 0% to 70%, accompanying by a red shift of maximum emission peak from 500 nm to 580 nm. Maybe the increasing intramolecular charge transfer (ICT) effect of TPAS-NO can explain this phenomenon (Gao et al., 2016). At 90%, the fluorescence intensity dramatically increased by 5.4 times, and reached its peak at 99% (24.1 times compared to 70%), which was attributed to the continued aggregation of TPAS-NO molecules, unambiguously demonstrating that TPAS-NO is a typical AIEactive molecule.

To evaluate the light-triggered total ROS production of TPAS-NO, 2′,7′-nonfluorescent Dichlorodihydrofluorescein diacetate (DCFH-Da) was employed as an indicator. As we can see, the fluorescence intensity rapidly increased along with the higher concentrations of TPAS-NO under a white light (20 mW/cm^2^) (Fig. 2D). Furthermore, the total ROS production of CS/CMC/TPAS-NO coating films were also accessed. The higher the concentration of TPAS-NO, the more ROS was produced, resulting in stronger fluorescence. This information suggests that our proposed CS/CMC/TPAS-NO coating film will be highly efficient in photodynamic inactivation.

### 3.4 Safety evaluation of TPAS-NO

#### 3.4.1 In vitro cytotoxicity

A red blood cell hemolysis experiment was conducted to evaluate the blood compatibility of TPAS-NO. As shown in **Fig. S4A**, the supernatant of the blank group (in deionized water) was dark red, indicating the rupture of mouse red blood cells. As the concentration of TPAS-NO increased, the hemolysis rate of mouse red blood cells slightly increased. Nevertheless, the hemolysis rate (4.97%) is still acceptable (≤ 5%) even at 200 μM TPAS-NO (Zeng et al., 2024), indicating excellent blood compatibility of TPAS-NO.

Hela cells and RAW 264.7 cells were used to evaluate the in cytotoxicity of TPAS-NO through CCK-8 experiment. As shown in **Fig. S4B**, the overall survival rate of both cells decreased with the increasement of TPAS-NO concentrations. However, the survival rate was no significant decrease in cell survival rate at TPAS-NO concentrations of 0–50 μM. Also, the survival rates were 86.7% and 84.5% for Hela and RAW 264.7 cells at 50 μM of TPAS-NO, suggesting that TPAS-NO is sufficiently safe (Jingxuan Sun, 2022; Taotao Zhou, 2019).

#### 3.4.2 In vivo cytotoxicity

The vivo cytotoxicity of TPAS-NO was evaluated through weight change, complete boold count (CBC), biochemical index and HE-stained organ slice of the mice before and after gavage. As shown in **Fig. S4C**, there are no significant differences in weighing blank, control and AIE groups of mice, indicating that the appetite of mice was not affected even gavage with AIE (50 μM TPAS-NO). To our delight, high concentrations of TPAS-NO (50 μM) didn't exerted any side-effects on the blood index or biochemical index on both female and male mice, as evidenced by the negligible differences among AIE group, blank group and control group (**Fig. S5**). This information reveals that the AIE-treated mice had no severe systemic inflammation or functional impairment of their livers and kidneys (Jingxuan Sun, 2022). Similarly, in the analysis of HE-stained organ (liver, spleen, lung, and kidney) slice, there are no abnormalities or lesions occurred (L. Luo et al., 2025) in the AIE-treated group (**Fig. S6**), as well as the blank and control group, indicating that TPAS-NO is sufficiently safe for further application.

### 3.5 Characterization of coating film

#### 3.5.1 Appearance and microstructure

As displayed in Fig. 3A, the CS/CMC/TPAS-NO coating films are transparent even at 7.2 μM of TPAS-NO (named CCT7.2), which is more favorable in practical food preservation compared with other natural, dark colored antibacterial agents (Dong et al., 2023). The surface and cross-sectional microstructures of CS, CC, CCT0.072, CCT0.72, and CCT7.2 coating films were also characterized by scanning electron microscopy (SEM). As can be seen in Fig. 3B, like CS and CC coating films, our CS/CMC/TPAS-NO coating films presented a relatively uniform and flat surface without obvious protrusions under SEM, suggesting a good mutual solubility among CS, CMC, and TPAS-NO. Insterestingly, CS coating film had some cracks attributed to the gaps between CS molecules. However, the structure of others film was more uniform in the cross-section, without obvious cracks because CS and CMC were connected by hydrogen bonding and electrostatic adsorption, making the structure of film more dense and uniform, which was similar with others research (Lee et al., 2023).

**Fig. 3:**
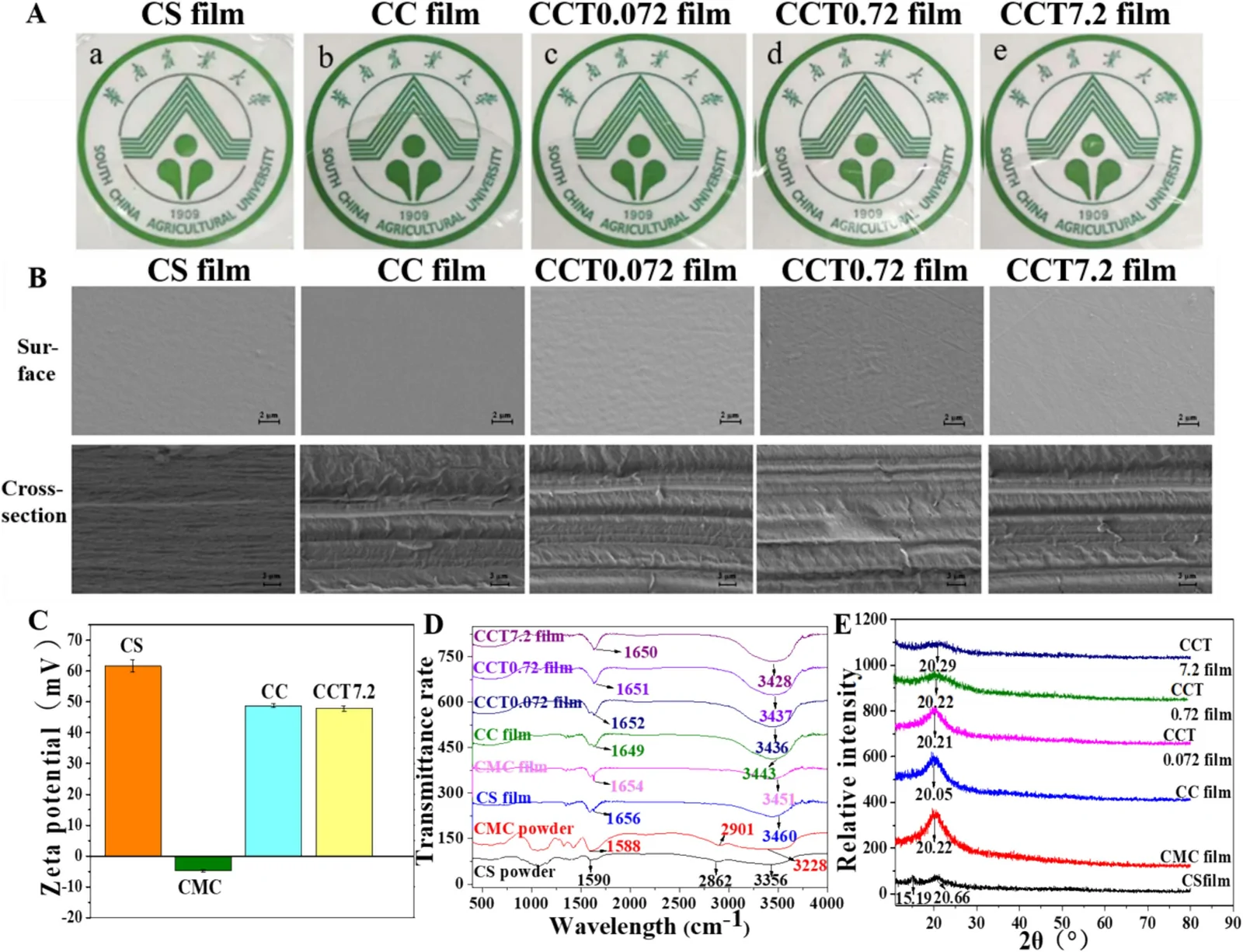
Characterization of this photosensitizers-loaded chitosan/carboxymethyl cellulose coating film, including (A) appearance, and (B) surface and cross-section of CS, CC, CCT0.072, CCT0.72 and CCT7.2 film by SEM, (C) zeta potential of coating film solution, (D) Fourier-Transform Infrared Spectroscopy (FT-IR) spectrum of coating film and powder, (E) X-ray diffraction (XRD) of coating film. (Each value is the mean of three replicate measurements, *n* = 3).

#### 3.5.2 Bonding mechanism

Given the opposite charges of CS and CMC, their combination is likely driven by strong electrostatic interactions. To confirm this, the zeta potentials of CS and CMC were tested to be 61.6 ± 2.0 mV and − 4.8 ± 0.3 mV (Fig. 3C). Maybe the amino group protonation of CS into and the carboxylic acid group dissociation of CMC can explain this. After mixing together, the zeta potentials were changed to be 48.7 ± 0.6 (CS + CMC, CC) and 48.1 ± 0.5 mV (CS + CC + TPAS-NO, CCT), demonstrating that CS and CMC were electrostatically adsorded and the TPAS-NO didn't affect the combination of CS and CMC.

In addition, FT-IR spectrum was employed to analyze the formation of covalent bonds or intermolecular forces between CS, CMC and TPAS-NO. As shown in Fig. 3D, CS and CMC powders exhibited broadband absorption at 3356 cm^−1^ and 3228 cm^−1^, corresponding to O—H and N—H stretching vibrations (Muhammed et al., 2023). At the coating film states, the CS and CMC still displayed broadband absorption at 3460 and 3451 cm^−1^, corresponding to the stretching vibration of O—H. After conjugation, the absorption peaks were enhanced and shifted to 3443 cm^−1^ (CC), 3436 cm^−1^ (CCT0.072), 3437 cm^−1^ (CCT0.72), and 3428 cm^−1^ (CCT7.2), respectively, indicating increasing hydrogen bondings among CS, CMC, and TPAS-NO molecules (Costa et al., 2021). On top of that, the pure CS film exhibited a characteristic peak at 1656 cm^−1^ (amide I band, C=O stretching), and the pure CMC film showed a peak at 1654 cm^−1^ (attributed to carboxylate/carbonyl stretching). Upon blending to form the CC film, this peak shifted to 1649 cm^−1^. This red shift (approximately 5–7 cm^−1^ toward lower wavenumbers) indicates the formation of strong intermolecular hydrogen bonding and electrostatic interactions between the protonated amino groups (-NH₃^+^) of chitosan and the carboxylate groups (-COO^−^) of CMC, which weaken the force constant of the C=O/C–O bonds. Interestingly, after incorporating increasing concentrations of TPAS-NO (CCT films), the peak gradually shifted back to higher wavenumbers, appearing at 1652, 1651, and 1650 cm^−1^, respectively. This progressive blue shift suggests that the introduction of TPAS-NO molecules partially perturbs or weakens the original CS-CMC interactions (likely through competitive hydrogen bonding or intercalation), thereby partially restoring the original vibrational frequency of the carbonyl groups.

The crystal structure of different coating film was evaluated by X-ray diffraction. As shown in Fig. 3E, the diffraction pattern of CS film exhibited broad and weak peaks at 2θ = 20.66° and 2θ = 15.19°, corresponding to crystal forms I and II. The CMC film exhibited a broad peak at 2θ = 20.22°, which was related to its relatively low crystal structure (Salama et al., 2019). Similarly, the CC, CCT0.072, CCT0.72, and CCT7.2 films exhibited broad peaks at 20.05°, 20.21°, 20.22°, and 20.29°, respectively, indicating their amorphous characteristics. Compared with the pristine CMC film, the gradual decrease in the intensities/areas of the peaks at around 15.96° and 25.67° in the CC, CCT0.072, CCT0.72, and CCT7.2 films indicates a progressive reduction in crystallinity. This phenomenon is likely attributable to the disruption of the original ordered packing of CMC chains upon incorporation of CS and TPAS-NO, suggesting that intermolecular interactions, probably involving hydrogen bonding and electrostatic forces, led to a more amorphous structure (Chen et al., 2025; Yanglin Wu, 2022). This interpretation is again supported by the above FTIR analysis (Fig. 3D), where shifts in O—H stretching vibrations were observed, corroborating the formation of hydrogen bonds among the three components.

#### 3.5.3 Thickness and light transmittance

As shown in Table 1, the thickness of the CC, CC0.072, CC0.72 and CCT7.2 coating films were almost the same, which is thicker than CS coating film due to fewer cracks and more uniform as well denser structure. All coating films had a high transmittance of about 90% in the visible light range of 400–800 nm (Table 1 **and Fig. S7**), which was consistent with the transparent appearance of the film. At 200–400 nm, the light transmittance of CCT0.072 (66.6%), CCT0.72 (59.5%) and CCT7.2 (46.6%) coating film was significantly lower than CS (81.4%) and CC (76.1%) coating film because of the p-π conjugation formed by the benzene ring of TPAS-NO structure (Li et al., 2022). These decreased transmittances in the UV region are beneficial for protecting food and reducing UV oxidation, delaying food spoilage.

**Table 1:** Thickness, transmittance at 300 nm (T_UV_), tensile strength (TS), elongation at break (EB), water content, water vapor transmittance rate (WVTR) and water contact angle of coating films (*n* = 3).

Coating film	Thickness (mm)	T_UV_ (%)	TS (MPa)	EB (%)	Water content (%)	WVTR (× 10^−11^ g·m^−1^·s^−1^·Pa^−1^)	Water contact angle (°)
CS	0.033 ± 0.003	81.4	5.86 ± 0.34	4.46 ± 0.56	37.02 ± 1.72	1.53 ± 0.04	29.42 ± 2.12
CC	0.042 ± 0.002	76.1	3.94 ± 0.32	23.01 ± 1.23	27.28 ± 1.12	1.02 ± 0.02	65.11 ± 2.33
CCT0.072	0.041 ± 0.004	66.6	5.04 ± 0.43	28.08 ± 1.42	28.21 ± 1.41	1.03 ± 0.01	65.67 ± 1.12
CCT0.72	0.043 ± 0.003	59.5	5.44 ± 0.32	29.65 ± 1.67	26.23 ± 0.92	0.98 ± 0.03	70.52 ± 1.13
CCT7.2	0.042 ± 0.001	46.6	6.2 ± 0.34	34.29 ± 0.54	25.12 ± 0.82	0.91 ± 0.02	78.48 ± 2.15

#### 3.5.4 Mechanical strength

The tensile strength (TS) and elongation at break (EB) were used to evaluate the mechanical performance of coating film. As shown in Table 1 **and Fig. S8**, the TS of CS coating film was lower than CC coating film due to the high crystallinity. With the addition of CMC, flexible segments (*β*-1,4-glycosidic bonds) and carboxymethyl groups were introduced, the rigidity of CS molecular chains was reduced, and the EB was improved. After the addition of TPAS-NO, the TS and EB were further improved since the TPAS-NO changed the conformation or arrangement between CS and CMC (Yang et al., 2024).

#### 3.5.5 Water content and water vapor transmittance rate

Lower water content is preferable in the preservation of fruits. As summaried in Table 1, CS coating film had higher water content than CC coating film because of the hydrophilic hydroxyl and amino groups (Chen et al., 2025). After the addition of higher concentrations of TPAS-NO, the water contents of coating films were further decreased.

The water vapor transmittance rate (WVTR) reflected the barrier property of the coating film against water vapor. As shown in Table 1, the CS coating film had higher WVTR than CC coating film, maybe the CS-CMC structure was denser than the pure CS molecules, which greatly limited the passage of water molecules. After the addition of TPAS-NO, the WVTR of coating films were decreased because the TPAS-NO could form hydrogen bonds with CS and CMC, resulting in a denser structure.

#### 3.5.6 Water contact angle

The bigger water contact angle, the poorer hydrophilicity of coating films, which is beneficial for extending the food shelf life (Liang et al., 2023). As shown in Table 1 **and Fig. S9**, after the addition of TPAS-NO, the water contact angle increased since TPAS-NO could promoted the connection between CS and CMC, making the structure tighter and less exposing hydrophilic groups to the outside of coating film.

### 3.6 ROS production and antibacterial performance of CS/CMC/TPAS-NO coating film

DCFH-Da was used to evaluate the ROS production of our CS/CMC/TPAS-NO coating film. As shown in Fig. 2D, the fluorescent intensity of blank film (CC film) was correlated with that of blank solution, avoiding the background interference. With the increasement of TPAS-NO content, more fluorescent DCFH molecules were generated, leading to more ROS. Importantly, compared with the liquid state TPAS-NO, our CS/CMC/TPAS-NO coating film yielded larger quantities of ROS, as evidenced by the stronger FL intensity of CCT0.72 film (0.72 μM) than 40 μM TPAS-NO solution.

To evaluate the antibacterial performance of our coating film, two Gram-negative bacteria (*E. coli and P. fluorescens*), two Gram-positive bacteria (*S. aureus and* L. *acidophilus*) and one fungus (*penicillium*) were selected. As we can see, without coating films and light irradiation, there were large quantities of bacterial colonies at each group (Fig. 4). Due to the antibacterial activity of CS (Luo et al., 2025), the bacteria growth was inhibited. Interestingly, the bacterial colonies of CCT72 film (72 μM TPAS-NO) without light irradiation were the same as the those in CC film, suggesting negligible dark toxicity of our CS/CMC/TPAS-NO coating films. Under light irradiation, the bacterial colonies were significantly reduced with the increased concentration of TPAS-NO. Especially, almost all kinds of selected microorganisms were killed by the CCT72 film, demonstrating robust yet broad-spectrum antibacterial performance of our proposed CS/CMC/TPAS-NO coating film.

**Fig. 4:**
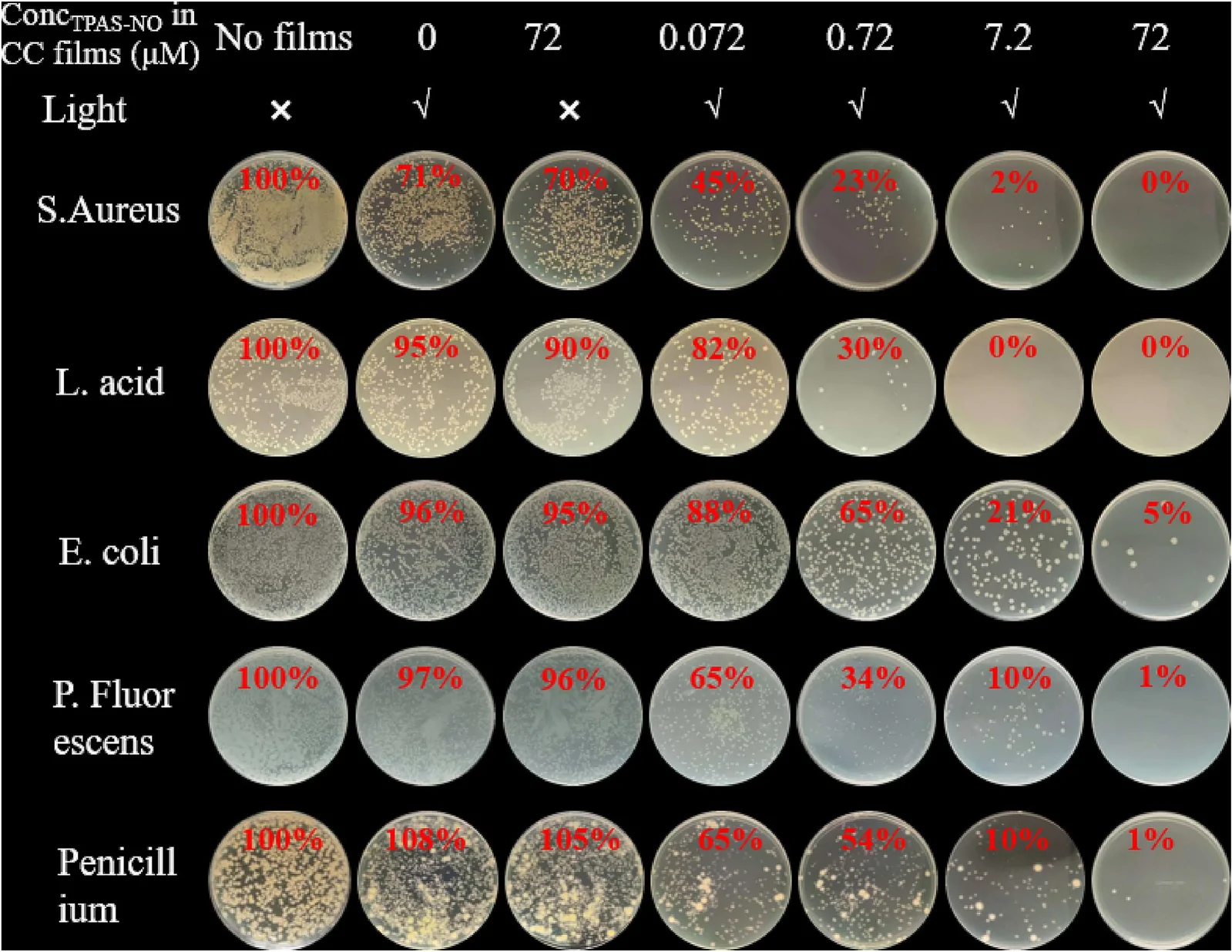
Antibacterial performance of the proposed photodynamic coating films at different TPAS-NO contents or different light irradiation, each value is the mean of three replicate measurements (The survival rates of different groups were marked in red). (For interpretation of the references to colour in this figure legend, the reader is referred to the web version of this article.)

### 3.7 Application of coating film solution in litchi preservation

#### 3.7.1 Appearance and browning index

Browning phenomenon will emerge during the preservation for litchi, resulting in a negative impact on the visual quality of litchi. As shown in Fig. 5 **A&B**, the litchis peels of blank group and CS coating films' group were rapidly turned brown and got moldy within four days. Under the protection of CC coating films, the peel browning and moldy outlooks were extended to six days. As what we expected, our CS/CMC/TPAS-NO coating films exhibited the best preservation performance, maintaining the high visual quality of litchi after eight days.

**Fig. 5:**
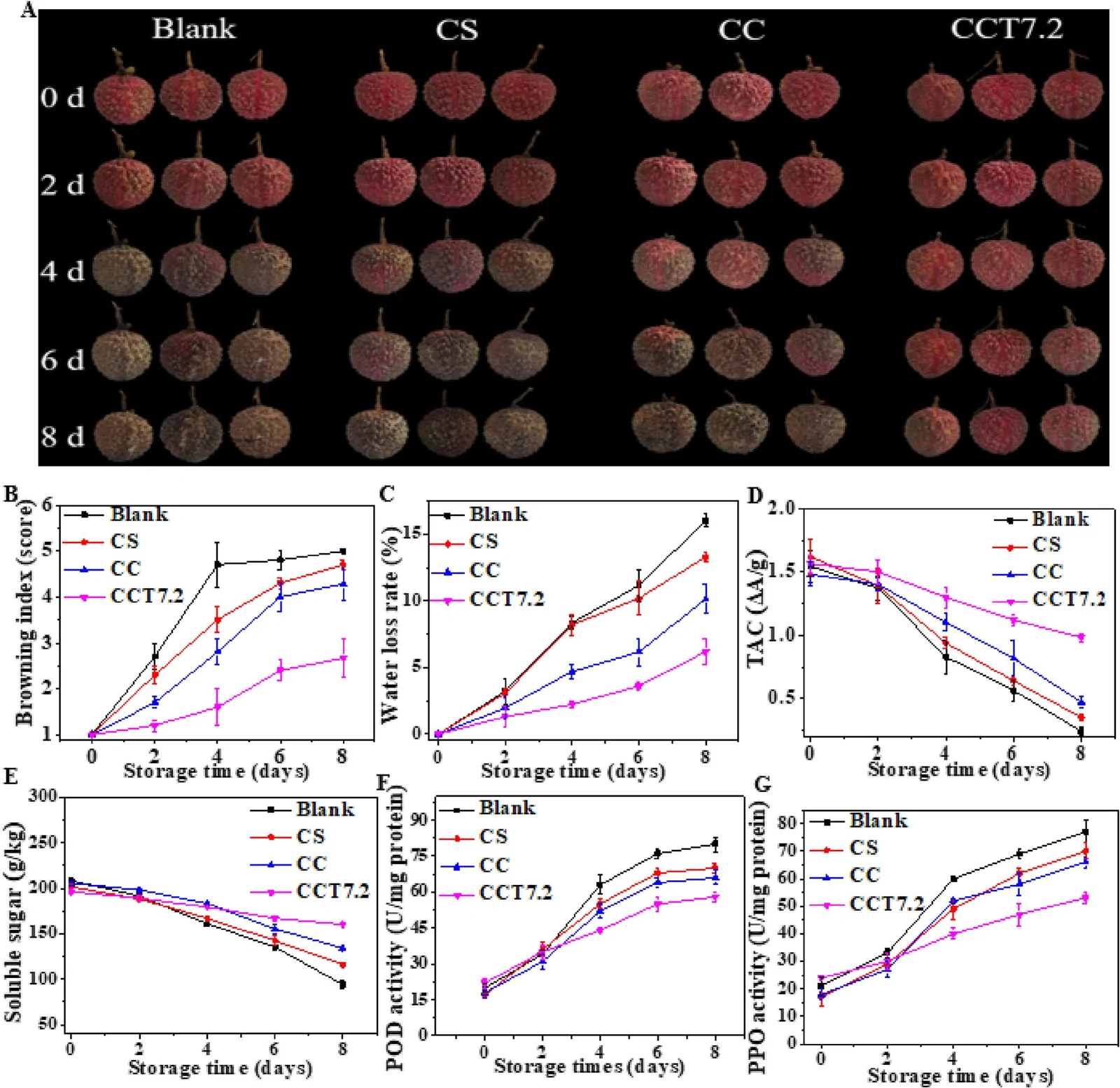
Preservation performance of this photodynamic coating films on litchi fruits, including (A) appearance changes (CC group: the litchi group preserved with CS/CMC coating film solution; CCT7.2 group: the litchi preserved with CS/CMC/TPAS-NO coating film solution); (B) browning index; (C) water loss rate; (D) total anthocyanin content (TAC); (E) soluble sugar content; (F) peroxidase (POD) activity; (G) polyphenol oxidase (PPO) activity of litchis in different groups. (Each value is the mean of three replicate measurements, *n* = 3).

#### 3.7.2 Water loss rate

Water loss affects the freshness of litchi. As shown in Fig. 5C, on the eighth day, the water loss rates of blank, CS, CC, and CCT7.2 group were 16.0%, 13.3%, 10.1%, and 6.2%, maybe low WVTR and excellent antibacterial performance of our CS/CMC/TPAS-NO coating films effectively reduced the water loss.

#### 3.7.3 Total anthocyanin content

Total anthocyanin content (TAC) is a key quality parameter for litchi, as it is susceptible to degradation caused by fruit respiration and microbial proliferation. Therefore, TAC serves as a reliable indicator of freshness, with higher values denoting better fruit quality. As shown in Fig. 5D, the CS/CMC/TPAS-NO coating film maintained an ultra-high TAC of 0.984 ΔA/g on day 8, substantially higher than that of the blank (0.243), CS (0.354), and CC (0.474) films. This significant difference demonstrates the superior preservative efficacy of the composite coating, which effectively suppresses respiration and microbial growth, thereby preserving anthocyanin integrity.

#### 3.7.4 Soluble sugar content

Soluble sugar content is an important indicator for evaluating the nutritional quality of litchi during preservation. On the 8th day, the soluble sugar contents of litchi in the blank, CS, CC, and CCT7.2 group were 94.2, 116.2, 134.3, and 160.4 g/kg (Fig. 5E), indicating that our CS/CMC/TPAS-NO coating film could effectively reduce the consumption of sugar substrates in litchis by slowing down the respiration and inhibiting the microorganisms growth.

#### 3.7.5 Peroxidase activity and polyphenol oxidase activity

Peroxidase (POD) can oxidize phenolic substances, exacerbating the peel browning of litchi. Polyphenol oxidase (PPO), similarly, catalyzes the oxidation of phenolics into brown pigments, leading to browning (Ali et al., 2018). As shown in Fig. 5F and Fig. 5G, the CS, CMC, and TPAS-NO treatments inhibited POD and PPO activities, with the CS/CMC/TPAS-NO composite coating exhibiting the lowest activities (58.3 and 53.5 U/mg protein, respectively) on day 8. This composite coating may alleviate peel browning through multiple mechanisms, it reduces structural damage, moderates respiration rates, minimizes water exchange with the external environment, decreases air oxidation of the peel, and suppresses microbial contamination. These combined effects contribute to the overall maintenance of fruit quality and extended shelf life.

## 4 Conclusion

In this study, CMC and photodynamic TPAS-NO (7.2 μM) were hybridized to functionalize the CS coating film, exhibiting high transparency, strong UB-blocking performance, outstanding mechanical strength, and acceptable hydrophobicity. In addition, our CS/CMC/TPAS-NO coating film also had excellent bactericidal effects on *Escherichia coli, Pseudomonas fluorescens, Lactobacillus acidophilus, Staphylococcus aureus and Penicillium*. Importantly, this coating film was further applied for the preservation of litchi fruits, which significantly delayed the pericarp browning, reduced water loss, diminished relative oxidase activity and preserved high levels of soluble sugar and total anthocyanin content for eight days. However, we recognize that the litchi samples may have been subjected to post-harvest treatments (e.g., fungicide dips or sulfur fumigation) before purchase, which could raise the baseline shelf life. In the next work, we need to validate it using freshly harvested lychees (or lychees with known preservative-free history) alongside commercially relevant benchmarks. It should be noted that the TPAS-NO was predominantly applied to the non-edible pericarp of litchi, with a working concentration of only 7.2 μM (approximately 7 times lower than the unequivocal safety margin established by the gavage assay (50 μM). Moreover, given that the edible aril is surrounded by a thick peel, any potential migration of the probe from the coating into the edible portion would be minimal. We believe that this unique, targeted, highly efficient and biocompatible photodynamic coating films can be extended to the preservation of more foods.

## CRediT authorship contribution statement

**Shuiyuan Liang:** Writing – original draft, Validation, Methodology, Investigation, Conceptualization. **Jiahui Guo:** Methodology, Investigation. **Xiyu Jiao:** Methodology. **Hongtao Lei:** Supervision. **Tian Guan:** Writing – review & editing, Supervision, Project administration, Funding acquisition, Conceptualization.

## Declaration of competing interest

The authors declare that they have no known competing financial interests or personal relationships that could have appeared to influence the work reported in this paper.

## Data Availability

Data will be made available on request.
