# Supplementary material for: Chitosan/Carboxymethyl cellulose edible coating loaded with aggregation-induced emission photosensitizers: A robust yet biocompatible antimicrobial film for Litchi preservation
Source: Food Chem X. 2026 Sep 19;39:104467. doi: 10.1016/j.fochx.2026.104467 (PMC13632095; doi:10.1016/j.fochx.2026.104467)
Supplement: Supplementary file 1 [file mmc1.docx]

***Supplementary Material***

**Non-toxic, antimicrobial aggregation-induced emission photosensitizers-loaded chitosan/carboxymethyl cellulose coating film for lychee preservation**

# Table of Content

1. Materials and methods

2. Supplementary figures

# 1. Materials and methods

## 1.1 Materials

Five strains, including positive bacteria (Lactobacillus acidophilus ATCC 4356, Staphylococcus aureus ATCC 6538), negative bacteria (Escherichia coli ATCC 8739, Pseudomonas fluorescens ATCC 13525) and fungus (Penicillium ATCC 9849) were purchased from the Wanjia Standard Material Research and Development Center (Henan, China). The Guiwei lychee fruits were brought from the supermarket in South China Agricultural University. The synthesized compounds were purified using column chromatography silica gel (200-300 mesh). All chemicals were commercial products and were used without any further purification.

## 1.2 Instrument

The molecular structure of synthesized compounds was confirmed by Nuclear Magnetic Resonance Spectra (NMR, BRUKER Germa ny). The excitement and emission spectrum was measured by fluorescence spectrophotometer (Yidian analysis, Shanghai). The microstructure, mechanical property, zeta potential, crystal structure, chemical bond and water contact angle of coating film were measured by Scan Electron Microscope (SEM, ZEISS-GeminiSEM 360 Germany), Electronic Universal Testing Machine (EUTM, Sansi technology China), Nano particle size and ZETA potential analyzer (Malvern, Britain), X-ray polycrystalline diffractometer (XRD, Nippon Science&Technology Co., Ltd), Fourier-Transform Infrared Spectroscopy (FT-IR, SHIMADZU-IRTracer-100) and Surface tension and surface contact angle tester (Dataphysics, Germany), respectively. The thickness of coating film was measured by Digital outer diameter micrometer (Alladin, Shanghai).

## 1.3 Theoretical calculation

All theoretical calculations referenced to the others methods[1] with some modifications and were run on Gaussian16 software. The ground (S_0_) and excited states (S_1_ and T_1_) were fully optimized by the DFT and B3LYP functional with the 6-31G’(d) basis set. In vibrational analysis calculations, all optimized structures in S_0_, S_1_, and T_1_ states were proved to be local minima without imaginary modes. The Gaussian output files were analyzed using Multiwfn[2] and VESTA software.

## 1.4 Synthesis of AIE photosensitizer TPAS-NO

### **1.4.1 The synthesis of 2-1 product**

The mixture of 4-(diphenylamino) phenyl) boronic acid (3 mmol), potassium carbonate (6 mmol) and 5-bromothiophene-2-aldehyde (4 mmol) were added into round bottom flask containing 16 mL tetrahydrofuran (THF): water (volume ratio, 3:1) solution. Then adding catalyst tetrakis (triphenylphosphine) palladium (0.1 mmol) and filling the round bottom flask with nitrogen to create a nitrogen reaction environment. Placing the round bottom flask in a constant temperature stirrer at 60 ℃ and stirring for 12 h. During the reaction, thin-layer silica gel chromatography (TLC) was used at irregular intervals to check whether the reaction was complete. The developing agent was petroleum ether: ethyl acetate (volume ratio, 4:1). After the reaction was complete, the reaction solution was extracted with ethyl acetate (EA) and water. The EA solution was taken with the drying of anhydrous sodium sulfate and then spinning the taken solution under reduced pressure to remove the organic solvent to get the crude product. Finally, the crude product was separated and purified by column chromatography, and petroleum ether (PE): EA (volume ratio, 5:1) was used as the eluent. Finally, the yellow solid 2-1 was obtained and dissolved with deuterated dimethyl sulfoxide (DMSO) to characterize the structure by NMR.

### **1.4.2 The synthesis of 2-2 product (TPAS-NO)**

The mixture of 2-1 (1 mmol) synthesized above, anhydrous potassium carbonate (2 mmol) and 2-methyl-5-nitrobenzoate (1.2 mmol) were added into a round bottom flask containing 20 mL anhydrous DMSO. The flask was placed in a constant temperature stirrer at 80 ℃ for stirring and reacting for 6 h. During the reaction, TLC was used to detect whether the reaction was complete at irregular intervals. The developing agent system was PE: EA (volume ratio, 7:3). After the reaction was complete, the reaction solution was extracted with EA and water. Collecting the solution of the EA layer and removing it by vacuum rotary evaporation to get intermediate product. Weighing the intermediate product (1 mmol) into round bottom flask containing 20 mL N, N-dimethylformamide (DMF), then 1-Ethyl-3-(3-dimethylaminopropyl) carbodiimide Hydrochloride EDCI (1.1 mmol) and N-Hydroxysuccinimide NHS (1.1 mmol) were added. After the reaction was complete, extracting the reaction solution with EA and water. Collecting the solution of the EA layer and removing it by vacuum rotary evaporation to get solid product. The solid crude product was purified by column chromatography, and the eluent was PE: EA (volume ratio, 4:1). The obtained product was freeze-dried at ultra-low temperature to obtain 2-2 product. Finally, the yellow solid 2-2 namely TPAS-NO was dissolved with deuterated DMSO to characterize the structure by NMR.

## 1.5 Safety evaluation of TPAS-NO

### **1.5.1 Mouse red blood cell hemolysis**

The mouse red blood cell hemolysis for TPAS-NO was processed based on others experimental protocol with some modifications [3]. Fresh mouse blood was added to anticoagulant and centrifuged at 8000 rpm for 10 min at 4 ℃. The supernatant was discarded, and 1 mL PBS was added to resuspend the precipitate to obtain a red blood cell solution. The solution was divided into 120 μL tubes and centrifuged at 8000 rpm for 10 min. The supernatant was discarded in advance, and 500 μL of deionized water was added PBS, PBS containing different concentrations of AIE photosensitizerTPAS-NO (1.56-200 μM) was left at room temperature for 6 h, centrifuged at 8000 rpm for 10 min, and the supernatant was taken to measure the absorbance at 540 nm. The hemolysis rate is calculated according to the following formula:

Hemolysis rate (%) = (OD_AIE_ - OD_PBS_) / (OD_Deionized water_ - OD_PBS_) × 100%	(1)

Among them, OD_AIE_ represents the absorbance value of the supernatant of the PBS group containing TPAS-NO in the centrifuge tube at 540 nm; OD_PBS_ represents the absorbance value of the supernatant from the PBS centrifuge tube at 540 nm; OD_Deionized water_ represents the absorbance value of the supernatant from the centrifuge tube of the deionized water group at 540 nm. Each value was measured in triplicate.

### **1.5.2 Hela cells and RAW 264.7 cells in vitro cytotoxicity**

Human cervical cancer cells (Hela cells) and macrophages RAW 264.7 were used to evaluate the cytotoxicity of AIE photosensitizer TPAS-NO, with some modifications made to the methods[4]. Firstly, Hela cells were cultured and passaged in Dulbecco's modified Eagle (DMEM) medium (10% FBS). 100 μL cell solution was added into a 96 well plate, ensuring that each well contains 2x10^4^ cells, and cultured overnight at 37 ℃ and 5% CO_2_ to allow the cells to adhere to the wall for growth. Then discarding the supernatant and adding DMEM (10% FBS) medium containing TPAS-NO of different concentrations (0-200 μM) for incubation for 4 h. Adding 10 μL CCK-8 solution to each well. After 30 min, reading the absorbance (OD) at 450 nm using a continuous wavelength multifunctional microplate detection instrument. Calculating the cell survival rate by comparing the OD of the cell group treated with TPAS-NO with the OD of the untreated cell group:

Cell survival rate (%) = (OD_untreated_ - OD_treated_) / OD_untreated_ × 100%	(2)

Among them, OD_untreated_ represents the absorbance value at 450 nm of the cell group without TPAS-NO and added CCK-8 solution for 30 min; The OD_treated_ represents the absorbance value at 450 nm of the cell group treated with TPAS-NO and added CCK-8 solution for 30 min. Each value was measured in triplicate.

### **1.5.3 Mouse in vivo cytotoxicity**

18 Kunming mice were enrolled, including 9 males and 9 females (divided into 3 groups, 3 mice each group, with Blank, Control, and AIE group for males and females respectively). For one week after enrollment, the mice were fed with normal food and water. After one week, all mice were fasted for 5 h before gavage. After 5 h, no gavage was performed as Blank group. 0.2 mL of physiological saline was administered using a syringe and gavage needle as Control group. 0.2 mL of physiological saline (containing 50 μM TPAS-NO) was administered to each mouse using a syringe and gavage needle as AIE group. The mice after gavage continued to raise for 10 days and feed them with food and water normally. At each time point, the mice were taken out and weighed. After 10 days, blood samples were taken from the eyeballs of the mice (followed by routine blood tests such as red blood cell count, white blood cell count, hemoglobin, lymphocytes, and neutrophils). The mice were euthanized using the neck cutting method or carbon dioxide asphyxiation method. The hearts, livers, spleens, kidneys, and lungs of the mice were dissected and removed (some of the livers and kidneys were cut out for biochemical indicators such as albumin, total protein, alanine aminotransferase, blood urea nitrogen, and glucose). The remaining organs were stored in 4% paraformaldehyde for further paraffin embedding, sectioning, and HE staining to observe the integrity of the organ tissues.

## 1.6 ROS production capacity of TPAS-NO

Firstly, 9.74 mg DCFH-DA powder was dissolved in DMSO to obtain 2 mM DCFH-DA solution, then adding 200 μL 2 mM DCFH-DA solution to the centrifuge tube containing 1.6 mL 0.01 M NaOH solution. After standing for 30 min in the dark, the solution was diluting to 10 mL with 0.01 M PBS buffer to obtain activated 40 μM DCFH solution. For the performance of TPAS-NO in generating ROS in solution, 150 μL TPAS-NO solutions (10, 20, 30, 40 μM) were mixed with 150 μL 40 μM DCFH solution and irradiating the mixed solution with white light for 20 min. The solution adding DMSO as the control group. For the performance of TPAS-NO in generating ROS in coating film: First, the DMSO and 40 μM DCFH solution were mixed in equal amounts to obtain a base solution. Then, sticking the coating film (diameter: 9 mm) onto the bottom of a 12 well plate, and adding 1 mL of base solution to 12 well plates. Placing the 12 well plate under white light for 20 min and the coating film without TPAS-NO as the control group. If ROS are generated in the solution, DCFH will be oxidized by ROS to DCF, which could emit fluorescence at 530 nm. Stronger fluorescence indicated the production of more ROS[5]. Finally, the fluorescence spectra of the above solution (λex= 488 nm, λem= 515-610 nm) were recorded to evaluate the ROS generation ability of the solution. Each value was measured in triplicate.

## 1.7 Characterization of coating film

The CS, CC, CCT0.072, CCT0.72, and CCT7.2 coating films covering the logo of South China Agricultural University were taken photo by using a Redmi K60 mobile phone to obtain the visible appearance of the films. The microstructure of the surface and cross-section of the coating film were observed by SEM.

## 1.8 Formation mechanism of the coating film

### **1.8.1 Zeta potential**

The Zeta potential difference of coating film solutions was measured by Nano particle size and ZETA potential analyzer. Each value was measured in triplicate.

### **1.8.2 XRD**

The crystal structure of the coating film was examined using an XRD in a scan range of 2θ from 5 to 80°.

### **1.8.3 FT-IR**

The intermolecular interaction of coating film was recorded by FT-IR spectrum in the range of 400-4000 cm^−1^ at a resolution of 2 cm^−1^ with 32 scans.

## 1.9 Characteristic of the coating film

### **1.9.1 Light transmittance**

The light transmittance of coating film in 200-800 nm was measured by UV-Vis[6]. Each value was measured in triplicate.

### **1.9.2 Thickness and mechanical property**

The thickness of the coating film was measured using a digital external micrometer, with an accuracy of 0.001 mm. Ten points on the film were selected for measurement and the average value was taken. The tensile strength (TS) and elongation at break (EB) of the coating film were tested using an electronic universal testing machine. Among them, the coating film was cut into strips (10 × 60 mm) and tested at an initial distance of 40 mm from the fixture and a speed of 1 mm/s. The TS and EB were calculated using the following equation:

TS (MPa) = F ÷ d × w	(3)

EB（%）= ∆L ÷ L_0_	(4)

Among them, F represented the maximum tension of the coating film at the time of fracture (N), d represented the thickness of the coating film (mm), w represented the width of the coating film (mm), ∆L represented the extension length of the coating film at the time of fracture (mm), and L_0_ represented the initial length between the fixtures (mm).

### **1.9.3 Water content and Water Vapor Transmission Rate (WVTR)**

The water content of CS, CC, CCT0.072, CCT0.72 and CCT7.2 coating film could be obtained by measuring the weight reduction after drying the coating film in 105 ℃ oven for 1 h. The WVTR of the coating film was slightly modified based on the standard method of ASTM E96-00: adding 30 mL of distilled water to a glass cup (diameter: 50 mm), then cutting the coating film into a disc larger than the diameter of the cup mouth and covering the cup mouth. The contact area between the coating film and cup mouth was completely sealed with Vaseline to prevent water vapor leakage through the gap. The initial weight of the cup covered with coating film was recorded and the cup was placed in a closed dryer containing 1000 g silica. The cup was taken out of the dryer every 2 h to weigh it until the weight stabilized before stopping the experiment. The WVTR was measured by using a linear regression equation between the weight loss of the cup and the time required for overall weight loss. The formula for measuring WVTR was as follows:

WVTR (g·m^-1^·s^-1^·Pa^-1^) = (dG × H) / (dt × dP × A)	(5)

Among them, dG represented the total weight lost by the glass cup (g), H represented the average thickness of the film (m), dt represented the time required for the glass cup to lose weight as a whole (s), dP= 2533Pa represented the partial pressure gradient, and A represented the area of water vapor passing through the film, that was, the area of the glass cup mouth (m^2^).

### **1.9.4 Water contact angel**

The water contact angle of coating film was measured and evaluated by Surface tension and surface contact angle tester. 3 μL deionized water was dropped onto surface of the coating film (20 × 20 mm) by using a microsyringe and taking photo to record the tangential angle between the water droplet and the surface of coating film after waiting 5 s. Finally, the tangential angle was measured as the water contact angle of coating film. Each value was measured in triplicate.

## 1.10 Lychee fruits preservation of the coating film

Lychee fruits were purchased from the supermarket on campus of South China Agricultural University and selected based on the screening criteria that 80% of the skin color was bright red and the weight size was similar to that observed by the naked eye. After cutting off the stems and rinsing with sterile water, the lychees were randomly divided into four groups. Three groups of lychees were soaked in CS, CC and CCT7.2 coating film for 5 min, and another group soaked in deionized water was used as blank group. Then, the lychees were dried at room temperature for 2 min and stored in 20 ℃ for 8 days (simulating cold chain transportation conditions[7]), taking out the relevant indicators for testing at different measurement day. In addition, the initial weight, total soluble particle size, and titratable acidity content of untreated lychee were 23.1 ± 1.5 g, 18.2 ± 0.2 °Brix, and 0.57 ± 0.04 g / 100g, respectively. Each value was measured in triplicate.

## 1.11 Fresh indicators of preserved lychee fruits

### **1.11.1 Appearance changes and browning index**

Appearance and color of lychee fruits during storage were marked by image acquisition with Redmi K60 mobile phone. The browning index was used to evaluate the degree of browning of lychee (evaluated as a score), with a score of 1 for non-browning lychee, 2 for 1-2 brown spots, 3 for some browning spots, 4 for 50% browning areas, and 5 for 75-100% browning areas[8]. Each indicator was measured in triplicate.

### **1.11.2 Water loss rate and Total anthocyanin content**

The weight loss rate of lychees during storage was measured by gravimetric method. The weight loss rate was calculated according to the following formula:

Water loss rate (%) = (W_1_, W_2_, W_3_…)/W_0_	(6)

Among them, W_0_ represented the initial weight of lychees after air drying, W_1_, W_2_, W_3_… represented the weight in different storage time (1,2,3… days).

The test method of total anthocyanin content referred to others’ method with some modification[9]. Taking 1 g lychee peel from each of the 10 lychees and cutting them into thin slices. The thin slices were extracted by 15 mL methanol solution containing 1% hydrochloric acid for 4 h, and then were filtered the solution to obtain the anthocyanin extract solution. The absorption values at 530, 620 and 650 nm of this solution were measured by a spectrophotometer. The total anthocyanin content (∆A) can be calculated using the following formula:

∆A·g^-1^ = (A_530_ - A_620_) - 0.1 (A_650_ - A_620_)	(7)

Among them, A_530_, A_620_, and A_650_ were the absorbance values of the filtered anthocyanin extract solution at 530, 620, and 650 nm, respectively.

### **1.11.3 Soluble sugar content**

The soluble sugar content of lychee was determined by anthrone sulfuric acid colorimetric method[10]. Firstly, different concentrations of glucose standard solutions (500, 400, 300, 200, 100, 80, 60, 40, 20, 0 μg/mL) and anthrone sulfuric acid solutions (100 mg anthrone dissolved in 100 mL of dilute sulfuric acid solution prepared by mixing 30 mL water and 76 mL concentrated sulfuric acid with a specific gravity of 1.84) were prepared (**Fig. S10**). Then, 1 g lychee flesh was added to a 10 mL centrifuge tube containing 8 mL 80% ethanol solution (a hole was opened in the tube cover with a 10 mL syringe needle), and the centrifuge tube was placed at 80 ℃ for 40 min to get the soluble sugar content extract solution. During this period, vortexes were taken out and mixed every 10 min. The mixture was centrifuged at 4000 rpm for 10 min, and the supernatant was taken out to dilute to 8 mL and dilute 100 times with 80% ethanol solution. Then, 200 μL diluent and glucose standard solution at different concentration was added to the centrifuge tube containing 1 mL anthrone sulfuric acid solution, respectively. Finally, the tubes were placed in boiling water for 10 min. After the solution cooled down, measuring the absorption value of solution at 625 nm. Finally, the glucose standard solution concentrations were as the x-axis and OD_625_ corresponding to concentration as the y-axis to draw a curve to determine the soluble sugar content in the sample diluent.

### **1.11.4 Activities of polyphenol oxidase (PPO) and peroxidase (POD)**

The methods for determining the activity of PPO and POD were based on the experimental methods of Ala et al. with slight modifications[11]. The determination of PPO activity: 1 g lychee peel was mixed with 10 mL 0.1 M citric acid solution (pH = 4, 1% PVP) for 2 min and incubating on ice for 30 min. The mixture was centrifuged at 10000 rpm for 5 min at 4 ℃. The supernatant was added to the centrifuge tube containing 1.45 mL 0.1 M citric acid solution (pH = 6.6) and 0.5 mL 100 mM catechol solution, shaking and measuring the absorbance at 420 nm. Enzyme activity was expressed as U/mg protein. Among them, every 0.01 unit increased in absorbance caused by PPO was equivalent to 1 active unit (U) of catalase. The determination of POD activity: 1 g lychee peel was mixed with 2 mL 0.1 M citric solution (pH = 4) and centrifuged at 10000 rpm for 10 min at 4 ℃ to obtain the supernatant. At the same time, the reaction solution was prepared by mixing 800 μL 50 mM PBS (pH = 5), 100 μL 40 mM hydrogen peroxide solution, and 100 μL 20 mM guaiacol solution and shaking well. Then, mixing the supernatant with the reaction solution in a 1:1 ratio and measuring the absorbance at 479 nm of the mixed solution. POD activity was expressed in U/mg protein, where a change of 0.01 units per min in absorbance represented the enzyme activity of one unit (U) of peroxidase. Each indicator was measured in triplicate.

# 2. Supplementary figures

## 2.1 The synthesis of TPAS-NO

### **2.1.1 The synthesis of 2-1**

Hydrogen spectrum data: 1H NMR (600 MHz, DMSO-d6) δ 9.86 (s, 1H), 8.00 (d, J = 3.9 Hz, 1H), 7.72 – 7.63 (m, 2H), 7.60 (d, J = 4.0 Hz, 1H), 7.35 (t, J = 7.7 Hz, 4H), 7.13 (td, J = 7.4, 1.1 Hz, 2H), 7.10 – 7.08 (m, 4H), 6.99 – 6.90 (m, 2H). Carbon spectrum data: 13C NMR (151 MHz, DMSO-d6) δ 183.69, 152.91, 148.55, 146.41, 140.86, 139.47, 129.76, 127.41, 124.97, 124.09, 123.97, 121.69. The attribution of hydrogen and carbon could indicate that 4- (diphenylamine) phenyl) boronic acid and 5-bromothiophene-2-aldehyde may be successfully linked together through the expected Suzuki-Miyaura coupling reaction in the presence of catalyst tetrakis (triphenylphosphine) palladium.

**Fig. S1** ^1^H- and ^13^C-NMR spectra of 2-1 product.

### **2.1.2 The synthesis of 2-2 (TPAS-NO)**

Hydrogen spectrum data: 1H NMR (600 MHz, DMSO-d6) δ 8.65 (s, 1H), 8.49 (s, 1H), 8.33 – 8.20 (m, 1H), 7.98 – 7.83 (m, 1H), 7.51 (d, J = 16.0 Hz, 2H), 7.36 (s, 1H), 7.07 (s, 3H), 6.94 (s, 6H), 6.82 – 6.66 (m, 2H), 2.91 (d, J = 27.0 Hz, 5H)。Carbon spectrum data: 13C NMR (151 MHz, DMSO-d6) δ 165.88, 164.86, 161.27, 159.76, 156.38, 148.31 (d, J = 16.2 Hz), 147.80, 146.27, 145.36, 144.60, 143.87, 141.06 – 137.62 (m), 134.13 (d, J = 80.7 Hz), 133.20, 132.96, 132.41, 128.92, 126.95, 121.99 – 120.40 (m), 114.22, 112.54, 20.89. The attribution of hydrogen and carbon could indicate that the 2-1 product and 2-methyl-5-nitrobenzoate may be linked together through the expected Knoevenagel condensation reaction under weakly alkaline conditions provided by anhydrous potassium carbonate, and the carboxyl group is activated into intermediate product 2-2 under the action of EDCI and NHS.

**Fig. S2** ^1^H- and ^13^C-NMR spectra of 2-2 product.

**Fig. S3** Emission spectra of TPAS-NO under different excitation wavelength.

**Fig. S4** The cytotoxicity of TPAS-NO: (A) Red blood cell hemolysis rate, (B) cell viability of Hela and RAW 264.7 cell of different TPAS-NO concentrations; (C) Body weight change of male and female mice after gavage. (Each value is the mean of three replicate measurements, n=3)

**Fig. S5** Blood index (A and B) and biochemical index (C and D) change of female and male mice without the gavage and with the gavage of Control and AIE. (WBC: white blood cell count; RBC: red blood cell count; HGB: hemoglobin; LYM: lymphocyte count; GRAN: neutrophil count; ALT: alanine aminotransferase; BUN: blood urea nitrogen; TP: total protein; ALB: albumin; GLU: blood glucose, each value is the mean of three replicate measurements, n=3)

**Fig. S6** HE-stained organ slice of male and female mice after gavage.

**Fig. S7** Light transmittance of different coating film.

**Fig. S8** Mechanical performance of different coating film. (Each value is the mean of three replicate measurements, n=3)

**Fig. S9** Water contact angle of different coating film. (Each value is the mean of three replicate measurements, n=3)

**Fig. S10** Glucose standard curve of different glucose concentration by anthrone sulfuric acid colorimetric method. (Each value is the mean of three replicate measurements, n=3)

**References**

[1] P. Yang, H. Huang, X. Xie, Removal of antibiotic resistant bacteria in wastewater by aggregation-induced emission photosensitizer, Environ. Pollut. 334 (2023) 121738.

[2] T. Lu, F. Chen, Multiwfn: a multifunctional wavefunction analyzer, J. Comput. Chem. 33 (2012) 580-92.

[3] L. Luo, W. Cai, W. Su, M. Wang, L. Zhang, J. Sun, J. Wang, Synergistic antimicrobial packaging film with photothermal-controlled biocatalytic capability for tangerine preservation, Food Hydrocolloids 160 (2025) 110832.

[4] R.H.L.W. Taotao Zhou, AIE conjugated polymer with ultra-strong ROS generation ability and great biosafety for efficient therapy of bacterial infection, Angew. Chem.-Int. Edit. (2019) 10-13.

[5] Y. Ning, S. Liu, Z. Ren, D. Yang, J. Li, L. Wang, An edible, photodynamic antimicrobial and fluorescent film containing aggregation-induced emission luminogens from berberine and citric acid for freshening cooked chicken, Food Hydrocolloids 155 (2024) 110206.

[6] S. Wang, R. Li, M. Han, D. Zhuang, J. Zhu, Intelligent active films of sodium alginate and konjac glucomannan mixed by Lycium ruthenicum anthocyanins and tea polyphenols for milk preservation and freshness monitoring, Int. J. Biol. Macromol. 253 (2023) 126674.

[7] S. Ali, A.S. Khan, A. Nawaz, M.A. Anjum, S. Naz, S. Ejaz, S. Hussain, Aloe vera gel coating delays postharvest browning and maintains quality of harvested litchi fruit, Postharvest Biol. Technol. 157 (2019) 110960.

[8] D. Sivakumar, L. Korsten, Fruit quality and physiological responses of litchi cultivar McLean's Red to 1-methylcyclopropene pre-treatment and controlled atmosphere storage conditions, Lwt - Food Science and Technology 43 (2010) 942-948.

[9] X. Zheng, S. Tian, Effect of oxalic acid on control of postharvest browning of litchi fruit, Food Chem. 96 (2006) 519-523.

[10] X. Su, H. Lin, B. Fu, S. Mei, M. Lin, H. Chen, Z. Zheng, H. Bo, D. Yang, Y. Lin, Egg-yolk-derived carbon dots@albumin bio-nanocomposite as multifunctional coating and its application in quality maintenance of fresh litchi fruit during storage, Food Chem. 405 (2023) 134813.

[11] S. Ali, A.S. Khan, A.U. Malik, M. Shahid, Effect of controlled atmosphere storage on pericarp browning, bioactive compounds and antioxidant enzymes of litchi fruits, Food Chem. 206 (2016) 18-29.
